# Cullin 3–Mediated Regulation of Intracellular Iron Homeostasis Promotes Thymic Invariant NKT Cell Maturation

**DOI:** 10.4049/immunohorizons.2300002

**Published:** 2023-03-23

**Authors:** Emily L. Yarosz, Ajay Kumar, Jeffrey D. Singer, Cheong-Hee Chang

**Affiliations:** * Immunology Graduate Program, University of Michigan Medical School, Ann Arbor, MI; † Department of Microbiology and Immunology, University of Michigan Medical School, Ann Arbor, MI; ‡ Department of Biology, Portland State University, Portland, OR

## Abstract

The E3 ubiquitin ligase cullin 3 (Cul3) is critical for invariant NKT (iNKT) cell development, as iNKT cells lacking Cul3 accumulate in the immature developmental stages. However, the mechanisms by which Cul3 mediates iNKT cell development remain unknown. In this study, we investigated the role of Cul3 in both immature and mature thymic iNKT cells using a mouse model with a T cell–specific deletion of Cul3. We found that mature iNKT cells lacking Cul3 proliferated and died more than wild-type cells did. These cells also displayed increased glucose metabolism and autophagy. Interestingly, we found that tight regulation of iron homeostasis is critical for iNKT cell development. Without Cul3, mature iNKT cells harbored higher levels of cytosolic iron, a phenotype associated with increased cell death. Taken together, our data suggest that Cul3 promotes iNKT cell development partially through intracellular iron homeostasis.

## Introduction

Invariant NKT (iNKT) cells comprise an innate-like lineage of T cells that recognize glycolipid Ags in the context of the MHC class I–like molecule CD1d. iNKT cell development depends on expression of the lineage-defining transcription factor promyelocytic leukemia zinc finger (PLZF) (1, 2). iNKT cells develop in the thymus, where they mature through four distinct stages: stage 0 (CD24^+^CD44^−^NK1.1^−^), stage 1 (CD24^−^CD44^−^NK1.1^−^), stage 2 (CD24^−^CD44^+^NK1.1^−^), and stage 3 (CD24^−^CD44^+^NK1.1^+^). Stage 0 and stage 1 iNKT cells are the most immature and have high proliferative capacity, glucose metabolism, and mitochondrial activity (3). In contrast, stage 3 cells are the most mature and are quiescent (3). Unlike conventional T cells, iNKT cells leave the thymus as effector cells capable of rapidly producing cytokines during infection (4). As such, iNKT cells are the first responders of the T cell–mediated immune response.

The E3 ubiquitin ligase cullin 3 (Cul3) plays a key role in several cellular processes, including antioxidation, cell cycle progression, and differentiation (5–9). Cul3 is known for its role in the Cul3-Keap1-Nrf2 trimeric complex, which controls cellular redox states (5). Additionally, Cul3 works with Cul1 to degrade cyclin E, modulating the transition from G_1_ to S phase in proliferating cells (7, 8). Cul3 also regulates neuronal development, adipogenesis, and myogenesis (6). However, the role of Cul3 in immunity is severely understudied.

Cul3 is a critical regulator of iNKT cell development, as iNKT cells lacking Cul3 fail to mature and acquire an effector phenotype (10). However, the mechanistic pathways by which Cul3 controls iNKT cell development remain unknown. Cul3 interacts with PLZF in the nuclei of thymic iNKT cells (10), but it is not known whether this interaction is important for iNKT cell development. In this study, we show that mature iNKT cells, defined as NK1.1^hi^ cells, proliferate and die more than wild-type (WT) cells in the absence of Cul3. Additionally, these cells display a metabolically active phenotype that is uncharacteristic of mature thymic iNKT cells. We also show that stage 3 iNKT cells lacking Cul3 exhibit iron overload, implicating Cul3 in iron homeostasis. Lastly, we show that Cul3 and PLZF appear to control iNKT cell development through independent mechanisms.

## Materials and Methods

### Mice

A mix of male and female mice ranging from 8 to 16 wk of age were used in all experiments. T cell–specific Cul3-deficient mice (Cul3^fl/fl^ CD4-Cre, referred to as Cul3 knockout [KO]) were generated by crossing Cul3^fl/fl^ mice (7) with CD4-Cre–expressing mice maintained in our colony. PLZF^−/−^ mice were generated by crossing PLZF^+/−^ parents. In experiments involving genetically modified mice, either Cul3^fl/fl^ or PLZF^+/+^ littermates (referred to as WT) were used as controls. C57BL/6 mice were used to study iron homeostasis over iNKT cell development. All mice were bred in-house and kept in specific pathogen-free conditions. All animal experiments were performed in accordance with the Institutional Animal Care and Use Committee of the University of Michigan.

### Cell isolation and staining conditions

Thymi were mechanically disrupted and transferred onto a 100-μm cell strainer to collect single-cell suspensions. Homogenized thymocytes were treated with 1.66% NH_4_Cl for 10 min to lyse RBCs, washed twice with 1× PBS, and resuspended in 1× PBS + 1% FBS (FACS buffer). iNKT cells were identified by flow cytometry by costaining with TCR-β and PBS-57–loaded CD1d tetramers in PE or allophycocyanin (National Institutes of Health Tetramer Core). Complete media, defined as RPMI 1640 medium supplemented with 10% FBS, 2 mM glutamine, and penicillin/streptomycin, served as the staining medium for several of the assays used in this study.

### Flow cytometry assays

The fluorescently conjugated Abs used for surface and intracellular staining in the presence of anti-FcγR mAb (2.4G2) were as follows: anti-mouse TCR-β (H57-597) allophycocyanin/Pacific Blue, anti-mouse CD4 (GK1.5) PerCP-Cy5.5/allophycocyanin-Cy7, anti-mouse CD8 (53-6.7) FITC/PE/PE-Cy7/Am Cyan, anti-mouse CD24 (M1/69) PE Texas Red, anti-mouse CD44 (IM7) FITC/PE/PerCP-Cy5.5, anti-mouse NK1.1 (PK136) PerCP-Cy5.5/PE-Cy7, anti-mouse CD71 (R17217) FITC/PerCP-Cy5.5, anti-Ki-67 (SolA15) PerCP-Cy5.5, anti-mouse PLZF (Mags.21F7) FITC/PE, anti-mouse RORγt (AFKJS-9) Pacific Blue, and anti-mouse T-bet (eBio4B10) PerCP-Cy5.5. Abs were purchased from eBioscience, BioLegend, or BD Biosciences.

For ferroportin (Fpn) staining, fixed cells were incubated with metal transporter protein Ab (rabbit anti-mouse MTP1/IREG1/Fpn) (Alpha Diagnostic) in FACS buffer. Ferritin, p62, and hexokinase II (HK2) expression were measured by anti-mouse ferritin (EPR3004Y) (Abcam), anti-p62 (sequestosome-1) (11C9.2) (MilliporeSigma), and anti-HK2 (EPR20839) (Abcam) staining, respectively, in cytoplasmic permeabilization buffer (BD Biosciences) after fixation. For ferritin and HK2, an AF488-conjugated anti-rabbit IgG secondary Ab (Invitrogen) was used. For p62, a PE-conjugated anti-mouse IgM secondary Ab (II/41) (Invitrogen) was used. Dead cells were excluded from the analysis based on propidium iodide (1 μg/ml), Live/Dead fixable aqua dead cell stain (Invitrogen), or Live/Dead fixable yellow dead cell stain (Invitrogen) signal. Data were acquired on a FACSCanto II (BD Biosciences) and analyzed using FlowJo v10.8.1 (Tree Star).

### In vivo BrdU incorporation

Eight- to 16-wk-old WT and Cul3 KO mice were injected twice i.p. (6 h apart) with 0.5 mg of BrdU (Sigma-Aldrich) in 0.2 ml of sterile 1× PBS. Approximately 18 h after the last injection, animals were euthanized, and single-cell suspensions were prepared from the thymi. Following surface staining, 3 × 10^6^ whole thymocytes were stained for BrdU incorporation using a BrdU flow kit (BD Biosciences) as per the manufacturer’s protocol.

### Annexin V staining

Following surface staining, 3 × 10^6^ whole thymocytes were washed with 1× annexin V binding buffer (BD Biosciences) and stained with PE-conjugated annexin V (Invitrogen) and PerCP-Cy5.5–conjugated 7-aminoactinomycin D (Invitrogen).

### Metabolic parameters

For each parameter, 3 × 10^6^ whole thymocytes were incubated with each of the following reagents as indicated. Cells were analyzed by flow cytometry following all metabolic stainings.

Total cellular reactive oxygen species (ROS) were measured by staining for 2′,7′-dichlorodihydrofluorescein diacetate (H_2_DCFDA) (1 mM) (Invitrogen). Cells were treated with H_2_DCFDA in complete media for 30 min at 37°C.

To measure glucose uptake, cells were incubated in 2-(*N*-(7-nitrobenz-2-oxa-1,3-diaxol-4-yl) amino)-2-deoxyglucose (2-NBDG) (Invitrogen) (20 µM) for 1 h at 37°C in glucose-free RPMI 1640 media containing 5% dialyzed FBS. HK2 expression was measured by incubating cells with HK2 Ab for 40 min at room temperature in the dark in cytoplasmic permeabilization buffer (BD Biosciences).

Mitochondrial membrane potential, mitochondrial mass, and mitochondrial ROS (mtROS) were measured using tetramethylrhodamine methyl ester perchlorate (TMRM) (60 nM) (Invitrogen), MitoTracker Green (30 nM) (Invitrogen), and MitoSOX (2.5 µM) (Invitrogen), respectively. Cells were treated with MitoTracker Green and TMRM for 30 min and MitoSOX for 25 min at 37°C.

### Labile iron pool

To measure the labile iron pool (LIP), 3 × 10^6^ thymocytes were stained with calcein-AM dye (0.02 µM) (Thermo Fisher Scientific) for 10 min at 37°C and analyzed by flow cytometry (excitation, 488 nm; emission, 517 nm). As calcein binds free iron, calcein fluorescence decreases. As such, LIP was calculated based on the ratio of calcein mean fluorescence intensity (MFI) of WT versus WT, WT versus Cul3 KO, or WT versus PLZF^−/−^ samples.

### Lipid peroxidation assay

Thymocytes (3 × 10^6^) were stained with 0.5× lipid peroxidation sensor (Abcam) for 30 min at 37°C according to the manufacturer’s instructions. Cells were then stained with surface markers in the presence of anti-FcγR mAb (2.4G2) and analyzed by flow cytometry.

### Statistical analysis

Graphs were prepared using GraphPad Prism software (version 9.2.0). Bar graphs showing relative data were prepared by dividing individual WT, Cul3 KO, or PLZF^−/−^ values by the average WT value for each tested parameter. For comparison among multiple groups, data were analyzed using a one-way ANOVA with a multiple comparison post hoc test. For comparison between two groups, either unpaired or paired Student *t* tests were used. A *p* value < 0.05 was considered statistically significant.

## Results

### Stage 3 iNKT cells exhibit a hyperproliferative phenotype in the absence of Cul3

**FIGURE 1.**
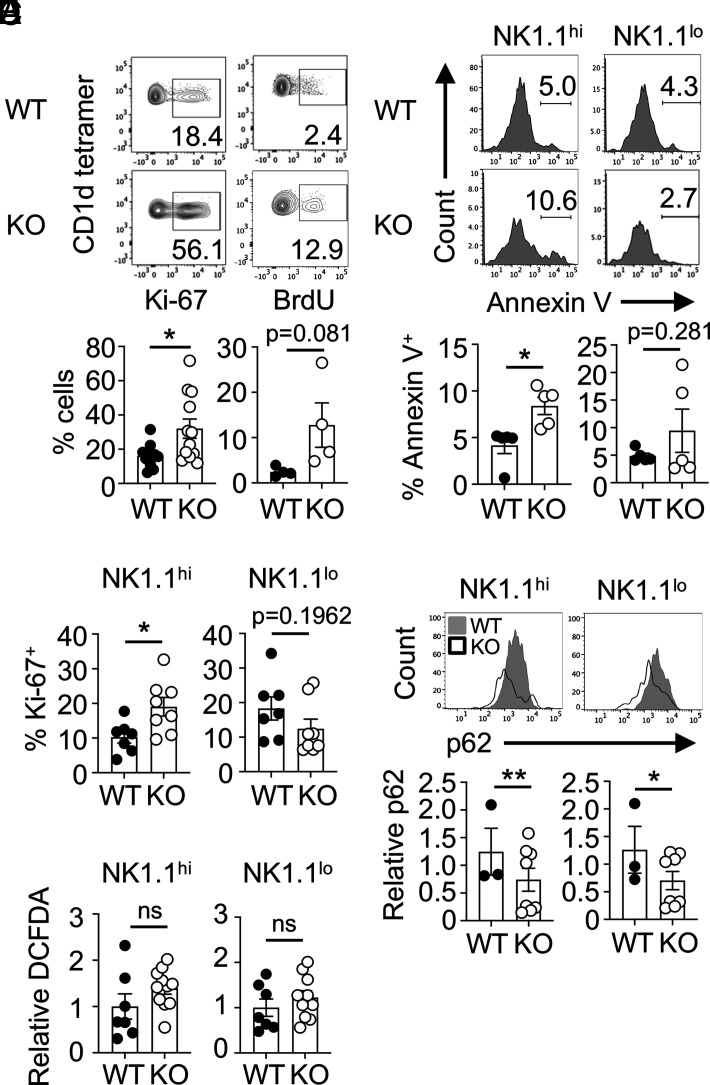
Cul3 inhibits developing iNKT cell proliferation and cell death. (**A**) Representative dot plots show the percentage of Ki-67^+^ and BrdU^+^ iNKT cells present in whole thymocytes. Bar graphs show pooled data for Ki-67 (*n* = 11 for WT, *n* = 12 for KO) and BrdU (*n* = 4). (**B**–**E**) Whole thymocytes from WT and Cul3 KO mice were stained for iNKT cell stagewise markers. (B) Bar graphs illustrate the pooled data for percentage of Ki-67^+^ cells in either the NK1.1^hi^ or the NK1.1^lo^ iNKT cell populations (*n* = 7 for WT, *n* = 8 for KO). (C) Representative histograms show percentage of annexin V^+^ thymic NK1.1^hi^ and NK1.1^lo^ cells from WT and Cul3 KO mice. Bar graphs show pooled percentage of annexin V^+^ cells (*n* = 5). (D) Representative histograms show expression of p62 in WT and Cul3 KO NK1.1^hi^ and NK1.1^lo^ cells. Bar graphs illustrate the MFI of p62 in Cul3 KO cells (*n* = 8) relative to WT cells (*n* = 3). Data are cumulative of two independent experiments. (E) Bar graphs show the MFI of H_2_DCFDA in Cul3 KO NK1.1^hi^ and NK1.1^lo^ cells relative to WT (*n* = 7 for WT, *n* = 10 for KO). Error bars represent mean ± SEM. *p < 0.05, **p < 0.005. ns, not significant.

Cul3 deficiency leads to a reduction in thymic iNKT cell frequencies and numbers (10). Additionally, progression through the cell cycle depends on the degradation of cyclins and other DNA replication components by Cul3 (7–9). These data suggest that Cul3-deficient thymic iNKT cells proliferate less than WT cells do. Therefore, we examined spontaneous proliferation by staining for Ki-67 in total thymic iNKT cells from either WT mice or mice having a T cell–specific deletion of Cul3 (Cul3 KO). Surprisingly, more Cul3 KO iNKT cells expressed Ki-67 than WT cells did (Fig. 1A, left panels). We also tested whether Cul3-deficient iNKT cells were actively proliferating in the thymus by examining BrdU incorporation. We found that thymic iNKT cells lacking Cul3 trended toward higher BrdU incorporation compared with WT cells (Fig. 1A, right panels). Taken together, these data indicate that Cul3 KO iNKT cells undergo uncontrolled proliferation in the thymus.

iNKT cells lacking Cul3 accumulate in the highly proliferative, immature stages of development (10, 11). Because Cul3 KO thymi contain more immature iNKT cells than WT thymi do (10), comparing total iNKT cell data is not appropriate. Therefore, we compared Ki-67 expression between NK1.1 lowly expressing (NK1.1^lo^) and NK1.1 highly expressing (NK1.1^hi^) thymic iNKT cells after excluding CD24^+^ cells from the analysis (Supplemental Fig. 1A, 1B), as Cul3 deficiency does not affect stage 0 iNKT cell numbers (10). As such, NK1.1^lo^ cells represent both stage 1 and stage 2 iNKT cells, whereas NK1.1^hi^ cells represent stage 3 iNKT cells. We found that more Cul3 KO NK1.1^hi^ cells expressed Ki-67 compared to WT cells (Fig. 1B, left panel, Supplemental Fig. 1C, top panels). In contrast, Cul3 KO NK1.1^lo^ cells showed a trend towards decreased Ki-67 expression (Fig. 1B, right panel, Supplemental Fig. 1C, bottom panels). These results suggest that Cul3 restrains proliferation primarily in stage 3 iNKT cells.

### Cul3 limits cell death in developing iNKT cells

Although thymic Cul3 KO NK1.1^hi^ iNKT cells proliferate more than WT cells do, their numbers are extremely low (10), indicating that Cul3 KO iNKT cells also die more than WT cells. Indeed, Cul3-deficient NK1.1^hi^ cells undergo more apoptotic cell death than do WT cells in the thymus (Fig. 1C). Autophagy mediates the transition of developing iNKT cells from being highly metabolically active to becoming quiescent (3). However, autophagy can also lead to cell death when left unchecked. Therefore, we asked whether thymic Cul3 KO iNKT cells exhibit increased autophagy. To test this, we measured levels of p62, which is degraded via autophagy (12). Therefore, high levels of p62 indicate low levels of autophagy, whereas low levels of p62 indicate high levels of autophagy. Both NK1.1^hi^ and NK1.1^lo^ Cul3 KO iNKT cells had lower levels of p62 than WT cells (Fig. 1D), indicating that autophagy is high in these cells.

Cul3 also regulates cellular ROS levels through its involvement in the Cul3-Keap1-Nrf2 trimeric complex. Loss of Cul3 could be particularly detrimental for iNKT cells, which acquire high levels of ROS as they egress to the periphery (13). In fact, dysregulation of the Cul3-Keap1-Nrf2 trimeric complex is detrimental to iNKT cell development (14). Therefore, we wondered whether the increased iNKT cell death in the absence of Cul3 is due to overactive ROS scavenging. However, total ROS levels were not different between WT and Cul3 KO iNKT cells in the thymus (Fig. 1E, Supplemental Fig. 1D), suggesting that Cul3 controls thymic iNKT cell death independently of Nrf2.

### Cul3 restricts glucose metabolism but not mitochondrial function in developing iNKT cells

**FIGURE 2.**
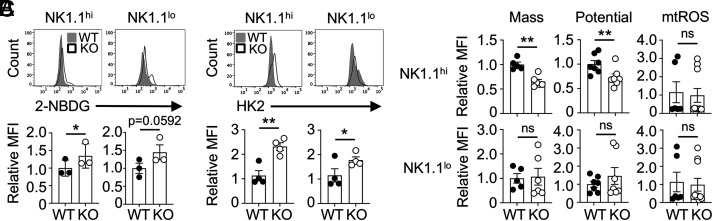
Cul3 primarily influences glucose metabolism in developing iNKT cells. (**A**) Representative histograms show glucose uptake in thymic NK1.1^hi^ and NK1.1^lo^ cells. Bar graphs show MFI values of 2-(*N*-(7-nitrobenz-2-oxa-1,3-diaxol-4-yl) amino)-2-deoxyglucose (2-NBDG) from Cul3 KO cells relative to WT cells (*n* = 3). (**B**) Representative histograms show expression of HK2 in NK1.1^hi^ and NK1.1^lo^ iNKT cells. Bar graphs illustrate the MFI of HK2 in Cul3 KO cells relative to WT cells from two independent experiments (*n* = 4). (**C**) Bar graphs show the MFI of MitoTracker Green (*n* = 5 for WT, *n* = 6 for KO), TMRM (*n* = 7), and MitoSOX (*n* = 6 for WT, *n* = 10 for KO) in Cul3 KO NK1.1^hi^ and NK1.1^lo^ cells relative to WT. Data for MitoTracker Green, TMRM, and MitoSOX are cumulative of two, three, and four independent experiments, respectively. Error bars represent mean ± SEM. *p < 0.05, **p < 0.005. ns, not significant.

iNKT cells in stage 0 use glycolysis to fuel their maturation; however, the resolution of this high rate of glycolysis is crucial for the development of functional stage 3 iNKT cells (15). Therefore, we hypothesized that NK1.1^hi^ iNKT cells lacking Cul3 would also display increased glucose metabolism. We found that glucose uptake was higher in NK1.1^hi^ iNKT cells (Fig. 2A). Although NK1.1^lo^ iNKT cells lacking Cul3 also trended toward higher glucose uptake, the data were not statistically significant (Fig. 2A). Expression of HK2, the first enzyme in the glycolytic pathway, was also higher in Cul3 KO iNKT cells compared to WT cells (Fig. 2B).

Peripheral iNKT cells use glucose to fuel the pentose phosphate pathway and mitochondrial metabolism rather than glycolysis (16). Mitochondrial activity is also high in the immature stages of iNKT cell development and resolves as thymic iNKT cells mature (15). To determine whether Cul3 regulates mitochondrial quiescence during iNKT cell development, we examined mitochondrial mass, mitochondrial membrane potential, and mtROS production in NK1.1^hi^ and NK1.1^lo^ iNKT cells. Cul3 KO NK1.1^hi^ cells had lower mitochondrial mass and membrane potential compared to WT cells despite producing similar levels of mtROS (Fig. 2C, top panels, Supplemental Fig. 2, top panels). In contrast, mitochondrial function was not affected by the loss of Cul3 in NK1.1^lo^ cells (Fig. 2C, bottom panels, Supplemental Fig. 2, bottom panels). Therefore, Cul3 may restrain glycolysis but support mitochondrial dynamics in developing iNKT cells.

### Cul3 prevents iron overload in stage 3 iNKT cells

**FIGURE 3.**
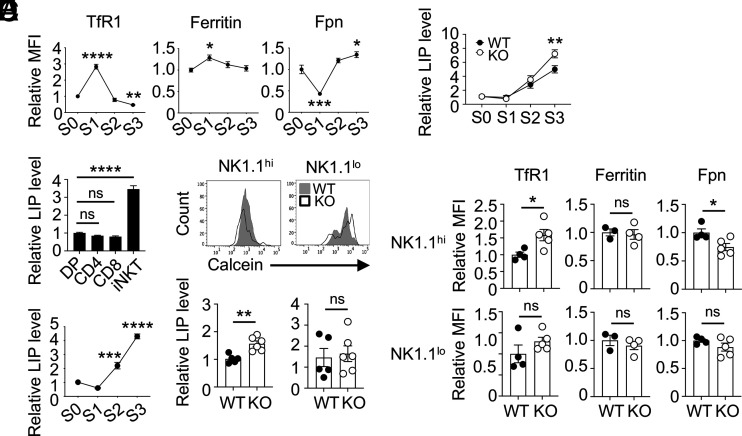
Cul3 modulates iron homeostasis during iNKT cell development. (**A**) Graphs show relative MFI values of TfR1, ferritin, and Fpn in B6 iNKT cells in the thymus. All data are relative to stage 0 (S0) cells (*n* = 5). (**B**) Bar graph shows labile iron pool (LIP) levels in thymic double-positive (DP), single-positive CD4, single-positive CD8, and iNKT cells from WT mice. LIP levels are relative to DP values (*n* = 5). (**C**) Graph shows the LIP level at each stage of development in B6 iNKT cells relative to S0 (*n* = 5). (**D**) Representative histograms depict calcein staining in WT and Cul3 KO NK1.1^hi^ and NK1.1^lo^ cells. Bar graphs show the LIP level in Cul3 KO cells (*n* = 6) relative to WT cells (*n* = 5) from three independent experiments. (**E**) Graph shows LIP levels in Cul3 KO (*n* = 7) iNKT cells at each stage of development relative to WT (*n* = 6) S0 cells. Data are cumulative of four independent experiments. (**F**) Graphs show MFI values of TfR1 (*n* = 4 for WT, *n* = 5 for KO), ferritin (*n* = 3 for WT, *n* = 4 for KO), and Fpn (*n* = 4 for WT, *n* = 5 for KO) in NK1.1^hi^ and NK1.1^lo^ cells lacking Cul3 relative to WT cells from three independent experiments. Error bars represent mean ± SEM. *p < 0.05, **p < 0.005, ***p < 0.0005, ****p < 0.0001. ns, not significant.

Poor mitochondrial function in Cul3 KO NK1.1^hi^ iNKT cells prompted us to investigate iron metabolism, as intracellular iron regulates CD4 T cell activation and proliferation by fueling the mitochondria (17). However, nothing is known about the role of iron in iNKT cell development and function. We began by measuring the levels of iron import, storage, and export proteins during stagewise development in C57BL/6 iNKT cells. Stage 1 iNKT cells, the most highly proliferative of all developing iNKT cells, showed the highest expression of the iron import protein transferrin receptor 1 (TfR1) as well as the iron storage protein ferritin (Fig. 3A, Supplemental Fig. 3A). In contrast, expression of the iron exporter Fpn is lowest in stage 1 iNKT cells (Fig. 3A, Supplemental Fig. 3A). TfR1 and ferritin levels decrease whereas Fpn levels increase through stages 2 and 3, largely returning to stage 0 levels (Fig. 3A, Supplemental Fig. 3A). These observations align with iNKT cells becoming quiescent as they mature. We also measured LIP in thymic iNKT cells, double-positive thymocytes, single-positive CD4 T cells, and single-positive CD8 T cells using calcein-AM dye. As calcein binds iron, the fluorescent signal is quenched, leading to a concomitant decrease in MFI. As such, high levels of calcein correlate with low cytosolic iron levels. We found that thymic iNKT cells have higher LIP levels than double-positive thymocytes, single-positive CD4 T cells, and single-positive CD8 T cells (Fig. 3B, Supplemental Fig. 3B). We also measured LIP at each stage of iNKT cell development. LIP is lowest in stage 1 iNKT cells (Fig. 3C, Supplemental Fig. 3A) but steadily increases as iNKT cells mature, with stage 3 cells harboring the highest LIP levels (Fig. 3C, Supplemental Fig. 3A). In all, our data show that iron is tightly regulated during iNKT cell development.

Given the antagonistic roles of Cul3 and iron on proliferation, we asked whether Cul3 modulates iNKT cell development by controlling iron homeostasis. We found that LIP is higher in Cul3-deficient NK1.1^hi^ iNKT cells but not NK1.1^lo^ iNKT cells compared to WT cells (Fig. 3D). Furthermore, stagewise analysis showed that LIP is higher in Cul3-deficient iNKT cells at stage 3 only (Fig. 3E). NK1.1^hi^ iNKT cells lacking Cul3 also displayed higher levels of TfR1, lower levels of Fpn, and similar levels of ferritin compared with WT cells (Fig. 3F, top panels, Supplemental Fig. 3C, top panels). In contrast, iron homeostasis in NK1.1^lo^ iNKT cells was not affected by the loss of Cul3 (Fig. 3F, bottom panels, Supplemental Fig. 3C, bottom panels).

Iron overload can lead to ferroptosis, a specialized form of cell death characterized by high cytosolic iron levels and rates of lipid peroxidation (18). We measured lipid peroxidation in Cul3 KO iNKT cells using a fluorescent sensor that is oxidized by cellular free radicals. As the sensor is oxidized, the fluorescent signal shifts from PE to FITC, allowing for detection via flow cytometry. The rate of lipid peroxidation within the cell can be determined by calculating the ratio of these two fluorescent signals, as a high FITC/PE ratio indicates high levels of lipid peroxidation. Unexpectedly, lipid peroxidation rates were similar in WT and Cul3 KO NK1.1^hi^ iNKT cells (Supplemental Fig. 3D), suggesting that these cells may die primarily by autophagy rather than ferroptosis. Taken together, our data indicate that Cul3 limits iron accumulation during iNKT cell development.

### Cul3 and PLZF play distinct roles during iNKT cell development

**FIGURE 4.**
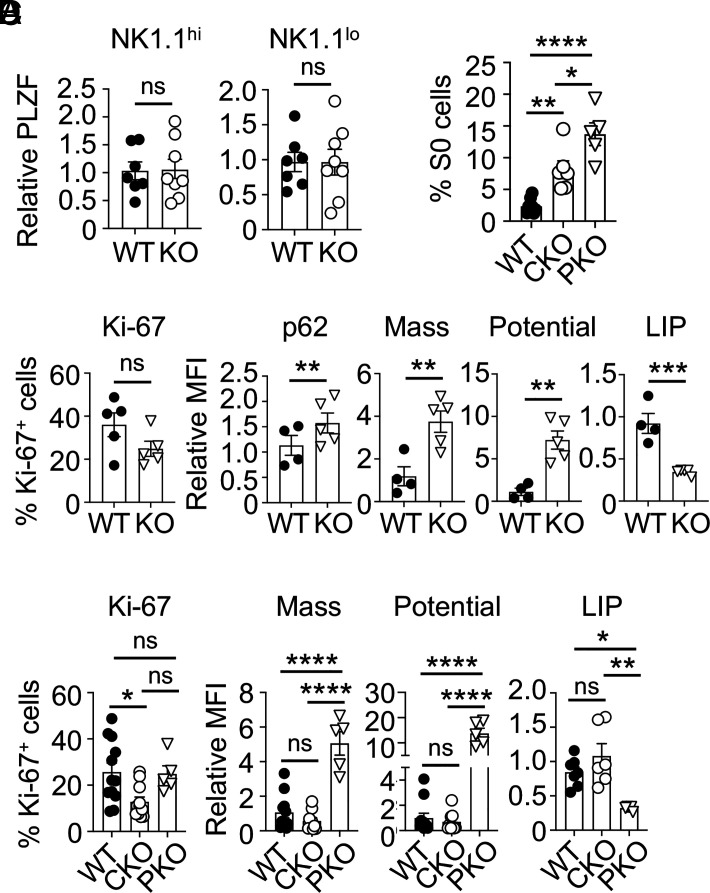
Cul3 and PLZF have distinct roles during iNKT cell development. (**A**) Bar graphs show the MFI of PLZF in Cul3 KO (*n* = 8) NK1.1^hi^ and NK1.1^lo^ iNKT cells relative to WT (*n* = 7). (**B**) Bar graph on the far left illustrates the percentage of Ki-67^+^ NK1.1^lo^ cells in whole thymocytes from PLZF^+/+^ (WT) and PLZF^−/−^ (KO) mice (*n* = 5). Other graphs show MFIs of p62, MitoTracker Green (mass), TMRM (potential), and calcein (LIP) in PLZF^−/−^ NK1.1^lo^ cells relative to WT (*n* = 4 for WT, *n* = 5 for KO). (**C** and **D**) WT cells are represented by filled circles, Cul3 KO (CKO) cells are represented by open circles, and PLZF^−/−^ (PKO) cells are represented by inverted open triangles. (C) Bar graph shows the percentage of stage 0 (S0) iNKT cells present in the thymi of WT (*n* = 8), Cul3 KO (*n* = 6), and PLZF^−/−^ (*n* = 5) mice. (D) Graphs show percentage of Ki-67^+^ cells (*n* = 11 for WT, *n* = 10 for CKO, *n* = 4 for PKO) as well as the MFIs of MitoTracker Green (mass; *n* = 11 for WT, *n* = 9 for CKO, *n* = 5 for PKO), TMRM (potential; *n* = 11 for WT, *n* = 9 for CKO, *n* = 5 for PKO), and calcein (LIP; *n* = 7 for WT, *n* = 6 for CKO, *n* = 5 for PKO) in Cul3 KO and PLZF^−/−^ NK1.1^lo^ iNKT cells relative to WT cells. Data in all panels are cumulative of at least three independent experiments. Error bars represent mean ± SEM. *p < 0.05, **p < 0.005, ***p < 0.0005, ****p < 0.0001. ns, not significant.

iNKT cells require PLZF to develop (1, 2). Cul3 and PLZF interact in the nuclei of iNKT cells, allowing Cul3 to ubiquitinate key epigenetic modifiers (10). However, PLZF can also be ubiquitinated (19), and PLZF levels steadily decrease as developing iNKT cells mature (1). We hypothesized that Cul3 ubiquitinates PLZF in iNKT cells, leading to proteasomal degradation of PLZF during iNKT cell development. As such, we expected that PLZF levels would be higher in Cul3 KO cells. However, PLZF levels were not affected by the loss of Cul3 (Fig. 4A, Supplemental Fig. 4A), indicating that Cul3 does not regulate PLZF expression.

iNKT cells differentiate into functional subsets during thymic development. Similar to conventional Th cells, these subsets can be defined based on their expression of the transcription factors PLZF, T-bet, and RORγt. NKT17 cells, which express an intermediate level of PLZF and high levels of RORγt, as well as NKT2 cells, which express the highest levels of PLZF, reach maturity at stage 2 of development (20). NKT1 cells, which express low levels of PLZF and high levels of T-bet, reach maturity at stage 3 of iNKT cell development (20). Therefore, most of the iNKT cells in a C57BL/6 mouse are iNKT1 cells (21). Because Cul3 deficiency inhibits iNKT cell development, we reasoned that iNKT subset distribution would be skewed in the absence of Cul3. Indeed, NKT1 frequencies were decreased whereas NKT2 and NKT17 frequencies were increased in the absence of Cul3 (Supplemental Fig. 4B), despite PLZF levels being unchanged (Fig. 4A, Supplemental Fig. 4A). These data suggest that changes in iNKT cell subset distribution in Cul3-deficient mice is due to iNKT cell developmental arrest rather than regulation of PLZF expression by Cul3.

To determine whether the interaction between Cul3 and PLZF is necessary for iNKT cell development, we examined iNKT cells from PLZF^−/−^ mice, which lack PLZF in all cells of the body. Loss of PLZF causes developing iNKT cells to accumulate in stage 1 (1, 2). As such, these mice lack mature NK1.1^hi^ iNKT cells. Therefore, our analysis of PLZF^−/−^ iNKT cells focused on the NK1.1^lo^ subset. PLZF deficiency did not affect proliferation (Fig. 4B, Supplemental Fig. 4C) in thymic NK1.1^lo^ iNKT cells. However, p62 levels were higher in PLZF^−/−^ NK1.1^lo^ cells (Fig. 4B, Supplemental Fig. 4D) compared with WT cells. Additionally, PLZF^−/−^ NK1.1^lo^ iNKT cells had higher mitochondrial mass and mitochondrial membrane potential than WT cells (Fig. 4B, Supplemental Fig. 4D). Lastly, PLZF^−/−^ NK1.1^lo^ iNKT cells harbor less LIP than WT cells do (Fig. 4B, Supplemental Fig. 4D).

Finally, we directly compared our Cul3 KO NK1.1^lo^ data with our PLZF^−/−^ NK1.1^lo^ data to attempt to dissect the roles of Cul3 and PLZF in iNKT cell development. The percentage of stage 0 cells in the thymus is a good indicator of aberrant iNKT cell development, since stage 0 cell frequencies increase as stage 3 cells die. PLZF deficiency led to a more severe block in iNKT cell development, as there was a higher percentage of stage 0 cells in PLZF^−/−^ thymi than in Cul3 KO thymi (Fig. 4C, Supplemental Fig. 4E). However, Cul3 appears to play a larger role than PLZF in controlling iNKT cell proliferation in stages 1 and 2, as only NK1.1^lo^ cells lacking Cul3 displayed a proliferative defect compared with WT cells (Fig. 4D). In contrast, PLZF appears to regulate mitochondrial function during the early developmental stages, as both mitochondrial mass and membrane potential are significantly higher in PLZF^−/−^ NK1.1^lo^ iNKT cells (Fig. 4D). PLZF also seems to control iron homeostasis early in iNKT cell development, as LIP levels were decreased in NK1.1^lo^ iNKT cells lacking PLZF but not Cul3 (Fig. 4D). In all, Cul3 and PLZF appear to control iNKT cell development largely via independent mechanisms.

## Discussion

In summary, our data indicate that Cul3 controls iNKT cell development by promoting quiescence during terminal maturation. To our knowledge, our study is also the first to show that iron homeostasis is dynamically regulated during iNKT cell development. Based on our data, we propose the following: iNKT cells may accumulate iron through transferrin receptor–mediated endocytosis, and this iron is retained during the course of iNKT cell development. To promote iron retention, Fpn levels drop in stage 1. As iNKT cells mature, Fpn levels rise. Maintaining high levels of Fpn may be beneficial to an effector cell by allowing for rapid iron flux after activation. These changes in iron homeostatic machinery during iNKT cell development prime stage 3 iNKT cells for rapid activation upon Ag encounter, allowing them to fulfill their role as effector cells.

Cul3-deficient NK1.1^hi^ iNKT cells exhibit increased proliferation, glucose uptake, and HK2 expression in comparison with WT cells. HK2 is a known target of Nrf2 (22), and Nrf2 regulates a variety of other glycolytic genes (23). Therefore, in the absence of Cul3, high Nrf2 levels may lead to the uncontrolled activation of glucose metabolism–related genes. iNKT cells lacking Keap1 also display increased glucose metabolism (14). Additionally, Keap1 deficiency leads to a block in iNKT cell development, which is rescued by T cell–specific deletion of Nrf2 (14). However, Nrf2 deficiency alone does not affect iNKT cell development (14). As such, high Nrf2 levels may be detrimental to iNKT cell development by preventing the transition from a metabolically active phenotype in stages 1 and 2 to a metabolically quiescent phenotype in stage 3.

Finally, our data suggest that PLZF and Cul3 control iNKT cell development through independent mechanisms. PLZF expression is tightly regulated during the course of iNKT cell development (1, 2), but whether Cul3 expression mirrors PLZF during stagewise development remains unknown. The lack of a specific Cul3 Ab prevented us from successfully investigating this question. PLZF^−/−^ mice lack virtually all mature iNKT cells (2), so comparing the roles of Cul3 and PLZF in the terminal stages of development was also not possible. Nonetheless, we compared NK1.1^lo^ cells from Cul3 KO and PLZF^−/−^ mice. We found that PLZF seems to drive the transition from stage 0 to stage 1, as PLZF deficiency led to a greater accumulation of cells in stage 0. PLZF also appears to regulate mitochondrial function and iron homeostasis in iNKT cells in stages 1 and 2. However, Cul3 seems to be the main regulator of proliferation during all stages of development. Cul3 also appears to restrict iron metabolism as developing iNKT cells reach maturity, as Cul3 KO NK1.1^hi^ cells harbor higher levels of LIP than WT cells do. In all, our study sheds new light on the roles of Cul3 and PLZF in iNKT cell development and maturation.

## Supplementary Material

Supplemental Figures 1 (PDF)
