# Supplementary material for: Cullin 3–Mediated Regulation of Intracellular Iron Homeostasis Promotes Thymic Invariant NKT Cell Maturation
Source: Immunohorizons. 2023 Mar 23;7(3):235–42. doi: 10.4049/immunohorizons.2300002 (PMC10122431; doi:10.4049/immunohorizons.2300002)
Supplement: Supplemental Figures 1 (PDF) [file IH_2300002_Supplemental_1.pdf]

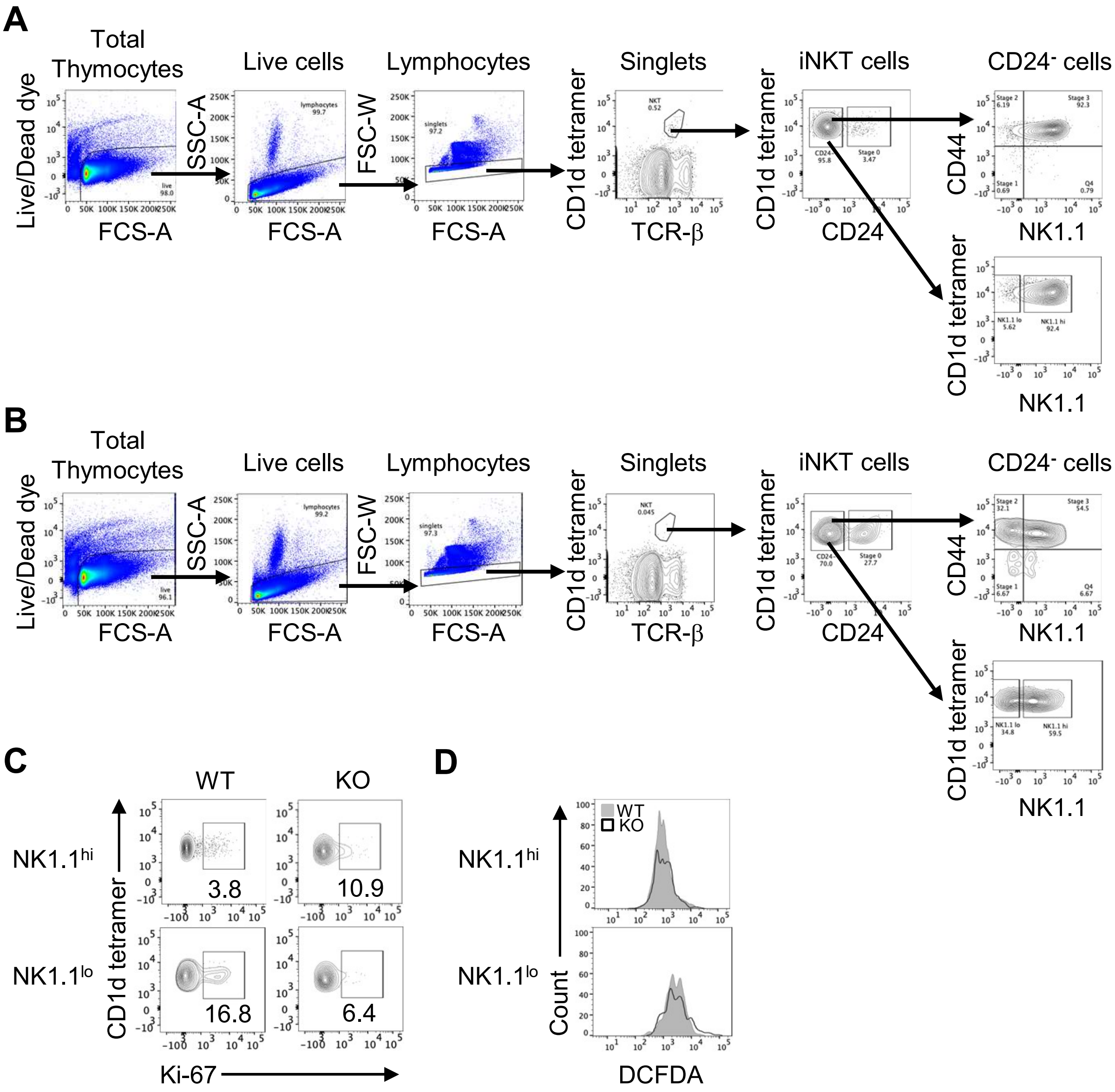

Supplemental Figure 1. (A-B) Gating strategy for stagewise iNKT cell analyses or NK1.1^{hi} and NK1.1^{lo} cell analyses from either WT (A) or Cul3 KO (B) mice. **(C-D)** Whole thymocytes from WT and Cul3 KO mice were stained for iNKT cell stagewise markers. **(C)** Representative dot plots illustrate the % Ki-67^{+} cells in either the NK1.1^{hi} or the NK1.1^{lo} iNKT cell populations (n=7 for WT, n=8 for KO). **(D)** Representative histograms show total ROS levels in NK1.1^{hi} and NK1.1^{lo} iNKT cells as measured by DCFDA staining (n=7 for WT, n=10 for KO).

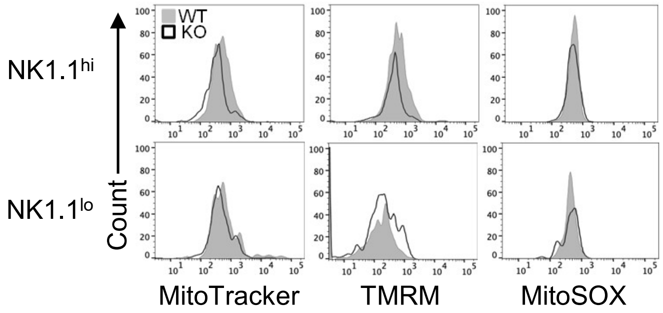

Supplemental Figure 2. Whole thymocytes from WT and Cul3 KO mice were stained for iNKT cell stagewise markers. Histograms show representative staining patterns for mitochondrial mass (n=5 for WT, n=6 for KO), mitochondrial membrane potential (n=7), and mitochondrial ROS production (n=6 for WT, n=10 for KO) in WT and Cul3 KO NK1.1^{hi} and NK1.1^{lo} cells as measured by MitoTracker^{TM} Green, TMRM, and MitoSOX staining, respectively.

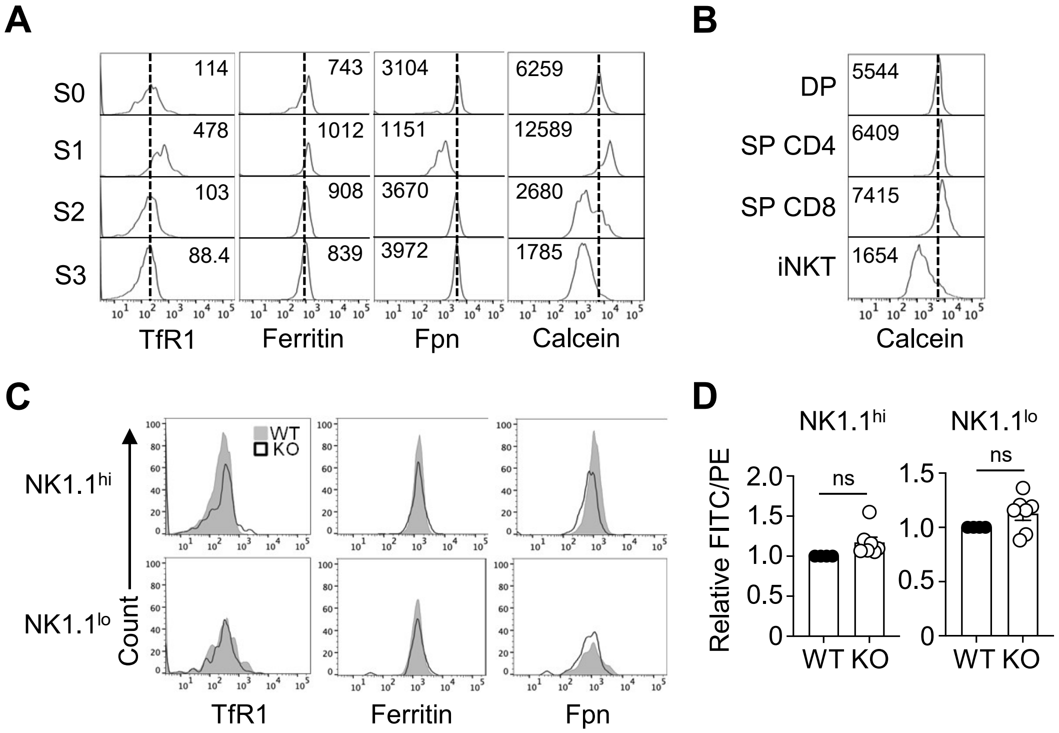

Supplemental Figure 3. (A) Histograms show the expression of transferrin receptor 1 (TfR1), ferritin, ferroportin (Fpn), and Calcein-AM dye in C57BL/6 iNKT cells at each stage of development (S0-S3). Numbers in each frame represent the mean fluorescent intensity (MFI) at each stage (n=5). **(B)** Representative histograms compare labile iron pool levels between WT thymic double-positive (DP), single-positive (SP) CD4, SP CD8, and iNKT cells as a function of Calcein-AM dye fluorescence. Numbers in each frame represent the MFI of Calcein in each cell type (n=5). **(C)** Overlaid histograms show the typical staining patterns of TfR1 (n=4 for WT, n=5 for KO), ferritin (n=3 for WT, n=4 for KO), and Fpn (n=4 for WT, n=5 for KO) in WT and Cul3 KO NK1.1^{hi} and NK1.1^{lo} iNKT cells. **(D)** Bar graphs show pooled lipid peroxidation levels as a function of FITC fluorescence divided by PE fluorescence in thymic Cul3 KO iNKT cells (n=7) relative to WT iNKT cells (n=4). Error bars represent mean ± SEM. ns: not significant.

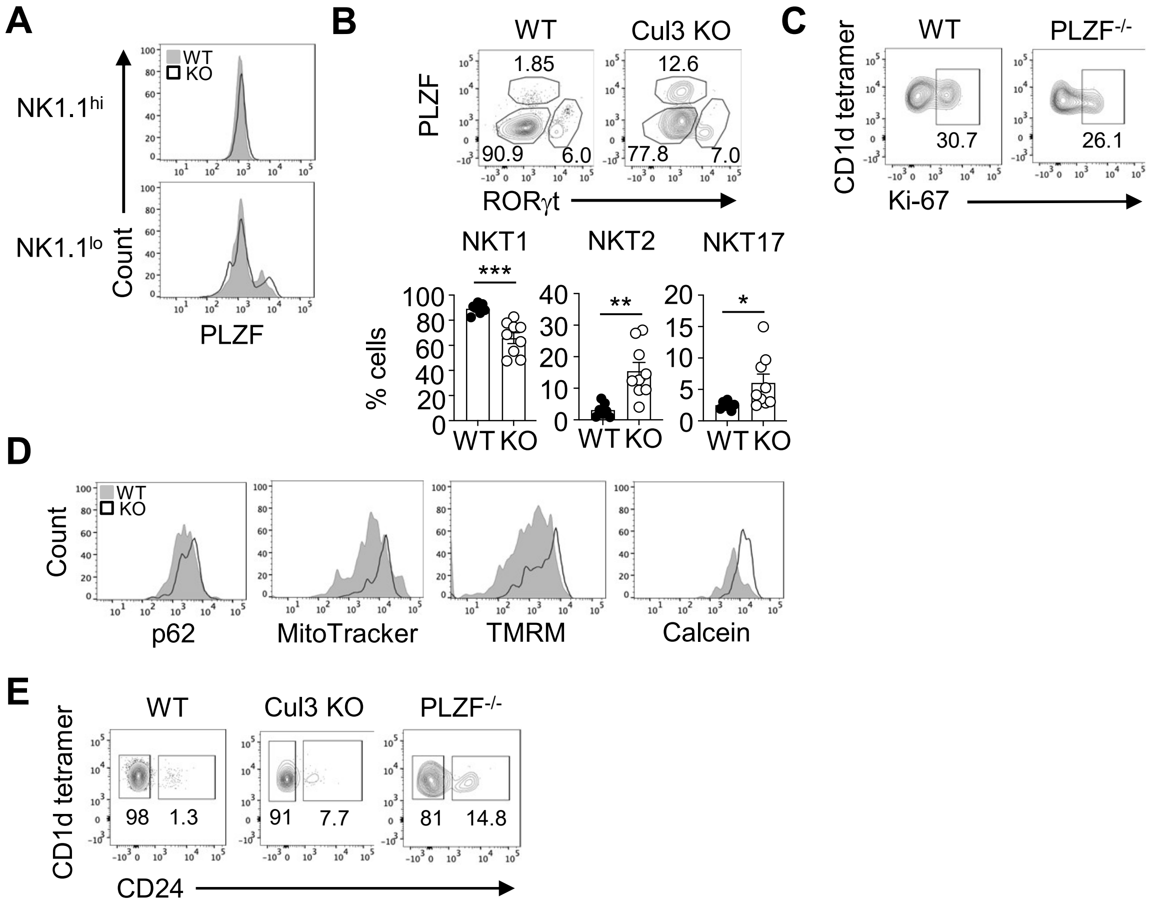

Supplemental Figure 4. (A) Representative histograms show PLZF levels in thymic NK1.1^{hi} and NK1.1^{lo} iNKT cells from WT (n=7) and Cul3 KO (n=8) mice. **(B)** Whole thymocytes from WT and Cul3 KO mice were stained for PLZF, T-bet, and RORγt. Representative dot plots show thymic iNKT cell subsets in WT (n=7) and Cul3 KO (n=9) mice. Bar graphs show the cumulative % of iNKT1, iNKT2, and iNKT17 cells in the thymi of mice from 4 independent experiments. **(C)** Representative dot plots illustrate the % Ki-67^{+} cells in NK1.1^{lo} iNKT cells from WT and PLZF$^{-/-}$ mice (n=5). **(D)** Representative histograms show the expression pattern for either p62, MitoTracker^{TM} Green, TMRM, and Calcein-AM dye in NK1.1^{lo} iNKT cells from WT and PLZF$^{-/-}$ mice (n=4 for WT, n=5 for KO). **(E)** Representative dot plots show the percentages of stage 0 (CD24^{+}) iNKT cells from WT (n=11), Cul3 KO (n=10), and PLZF$^{-/-}$ (n=4) mice. Error bars represent mean ± SEM. *p<0.05, **p<0.005, ***p<0.0005.
